# Prevalence of Histopathologic Types of Gingival Lesions in the Iranian Population: A 22‐Year Retrospective Study

**DOI:** 10.1002/cre2.911

**Published:** 2024-06-16

**Authors:** Nafiseh Shamloo, Mostafa Alam, Armin Khaleghi

**Affiliations:** ^1^ Department of Oral and Maxillofacial Pathology, School of Dentistry Shahid Beheshti University of Medical Sciences Tehran Iran; ^2^ Department of Oral and Maxillofacial Surgery, School of Dentistry Shahid Beheshti University of Medical Sciences Tehran Iran; ^3^ Dental Research Center, Research Institute of Dental Sciences, School of Dentistry Shahid Beheshti University of Medical Sciences Tehran Iran

**Keywords:** biopsy, gingiva, mouth, peripheral giant cell granuloma

## Abstract

**Objectives:**

Gingiva is one of the supporting tissues around the teeth that can be affected by various neoplastic or nonneoplastic lesions. Previous studies have examined several types of gingival lesions, but the lack of a standardized classification system has hindered meaningful comparisons. Additionally, many studies focused primarily on reactive lesions. Our study aims to contribute to the understanding of gingival lesions by investigating their prevalence across age groups, genders, sites, and by their clinical presentation. This research could lead to improved diagnostic accuracy and treatment strategies.

**Materials and Methods:**

This retrospective study explores the prevalence of gingival lesions based on biopsies during a 22‐year span. The patient's demographic details, including age, gender, and lesion's clinical presentation were systematically collected. These lesions were categorized into six groups. Descriptive statistics, *χ* ^2^ test of independence, and one‐way ANOVA were used for data analysis.

**Results:**

Among the 7668 biopsied lesions, 684 (8.9%) lesions were located in the gingiva, with a greater occurrence in women (63.5%). Soft tissue tumors represented the most prevalent group in the gingival lesions (72.1%), and peripheral giant cell granuloma (PGCG) was the most frequent lesion (21.2%), followed by, pyogenic granuloma (19.3%), peripheral ossifying fibroma (17.8%) and focal fibrous hyperplasia (7.6%); all of which predominantly affected women, with mean ages falling in the fourth decade of life. Squamous cell carcinoma was recognized as the most common malignancy.

**Conclusion:**

In this study, PGCG was found to be the most common lesion in the gingiva in Iranian population. Further analysis using a unanimous categorization is required to confirm these results.

## 1 Introduction

Gingiva is one of the supporting tissues around the teeth, covering the alveolar bone underneath (Alblowi and Binmadi 2018). Most gingival lesions are formed by periodontal diseases originating from dental plaques. However, some types of neoplastic and nonneoplastic lesions also involve the gingiva with etiologies other than bacterial plaques (Gupta et al. 2022; Li et al. 2021; Montazer Lotf‐Elahi, Farzinnia, and Jaafari‐Ashkavandi 2022). Neoplastic growths exist in benign or malignant forms determined by their progressive expansion (Gupta et al. 2022). In a study conducted in 2016, gingiva was found to be the most common site for SCC and hyperkeratosis in the oral cavity, ranking as the second most common site after the tongue for dysplastic changes (Martínez et al. 2016). Nonneoplastic lesions, on the other hand, are usually emerge as a reactive response to various chronic irritations, such as focal fibrous hyperplasia (FFH), pyogenic granuloma (PG), peripheral giant cell granuloma (PGCG), and peripheral ossifying fibroma (POF) (Montazer Lotf‐Elahi, Farzinnia, and Jaafari‐Ashkavandi 2022; Dutra et al. 2019).

To our understanding, several studies examined all types of gingival lesions (Alblowi and Binmadi 2018; Gupta et al. 2022; Li et al. 2021; Montazer Lotf‐Elahi, Farzinnia, and Jaafari‐Ashkavandi 2022; Tamiolakis et al. 2018; Manjunatha et al. 2014; Hernández‐Ríos et al. 2018; Kamath, Vidya, and Anand 2013; Carbone et al. 2012; Shamim et al. 2008; Orikpete and Iyogun 2021). Yet the absence of a consensus on classification posed challenges in comparing the findings. Also, a lot of studies were only limited to reactive lesions which were comprising the majority of gingival lesions.

A few epidemiologic studies regarding gingival lesions have been reported in Iran. In this study, we evaluated the prevalence and distribution of gingival lesions in an Iranian population during a 22‐year period between 2001 and 2022.

## 2 Methods

In this retrospective study, oral biopsies were collected during a 22‐year period (2001–2022) from archive of oral and maxillofacial pathology department, Shahid Beheshti dental school, Tehran, Iran. Gingival lesions were analyzed across age, gender, size, type of lesion, and clinical presentation.

Reports with incomplete or ambiguous diagnoses, duplicate entries, and also lesions on alveolar mucosa were excluded from the study. These criteria were applied to enhance the accuracy of the study.

The lesions were categorized into the following six groups: epithelial lesions, soft tissue tumors, inflammatory lesions, dermatologic diseases, hematologic disorders and Peripheral odontogenic tumors.

The collected data were analyzed using SPSS 26 software. Descriptive statistics were used to report the prevalence of biopsied gingival lesions across age, gender, type of lesion, and by their clinical presentation. Continuous variables were expressed as mean ± SD. Also, the *χ* ^2^ test of independence and one‐way analysis of variance were used to analyze the association of the lesions with categorical and continuous variables, respectively. Results were considered statistically significant when the *p* value was less than 0.05. This study was approved by the ethical review Committee (IR.SBMU.DRC.REC.1400.144). This research is based solely on archived data, and no personal information of the patients has been utilized or disclosed.

## 3 Results

Throughout the 22‐year span of this study, 684 gingival lesions (8.9%) were found among a total of 7668 cases, with 250 cases in males (36.5%) and 434 cases in females (63.5%). The mean age of the individuals with gingival lesions was 37 ± 18.9 years. The oldest diagnosed case was a 97‐year‐old woman with epithelial hyperplasia with dysplasia, while the youngest was a 9‐month‐old infant girl diagnosed with langerhans cell histiocytosis. The 20–39 years age group exhibited the highest prevalence of gingival lesions, totaling 230 cases (37%).

**Figure 1:**
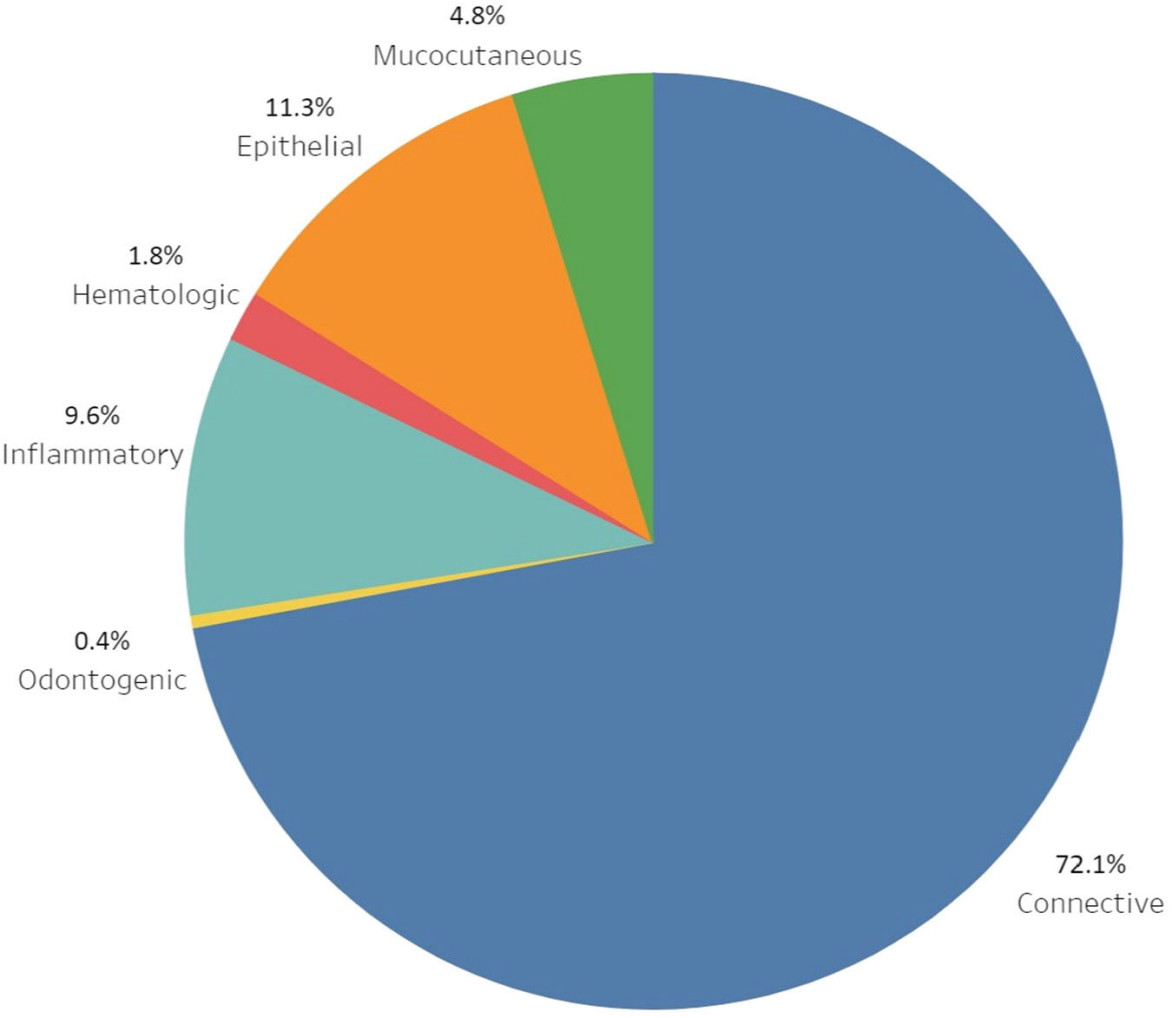
Distribution of gingival lesions across groups.

**Figure 2:**
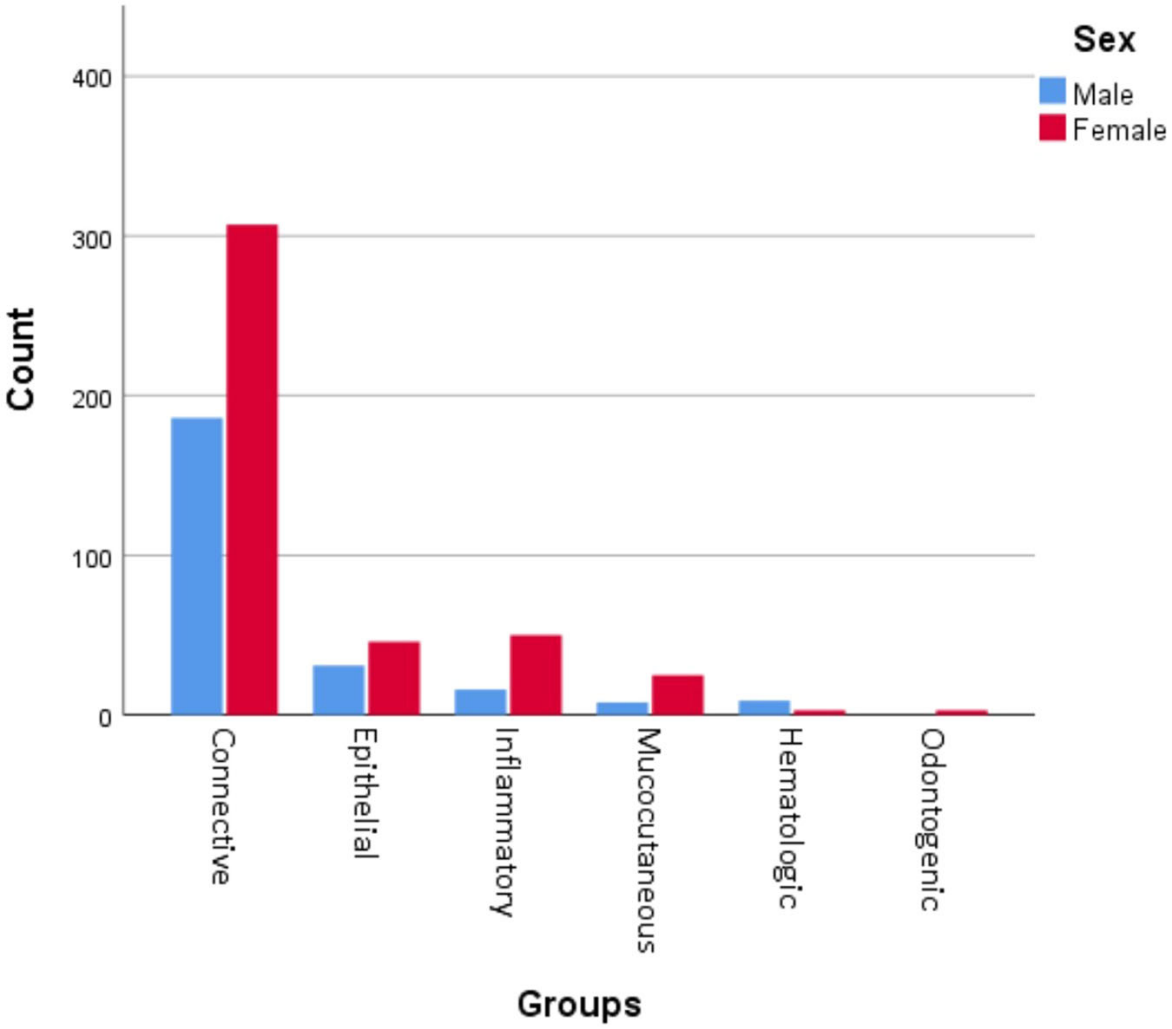
Distribution of gingival lesions based on gender.

Figure 1 shows the distribution of gingival lesions. Out of these 684 lesions, soft tissue tumors had the highest prevalence with 493 cases (72.1%). Following that, epithelial lesions accounted for 77 cases (11.3%), and inflammatory lesions represented 66 cases (9.6%), respectively. As illustrated in Figure 2, lesions were more frequently observed in women, except for hematologic disorders, which were more prevalent in men (*p* = 0.012).

**Figure 3:**
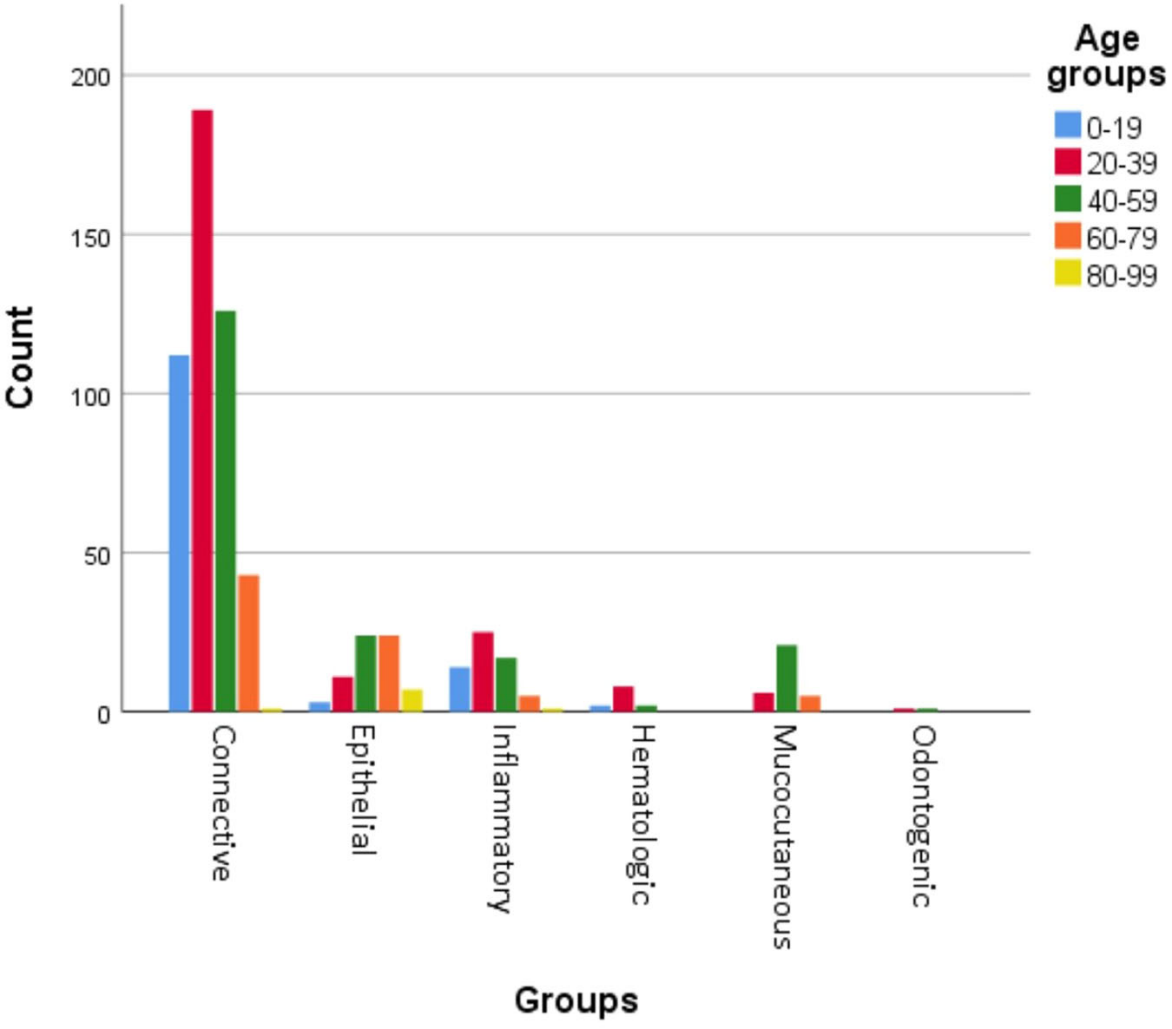
Prevalence of gingival lesions in age groups.

Soft tissue tumors displayed higher occurrence within the 20–39 age group (*p* < 0.001) whereas dermatologic diseases were predominantly among individuals aged 40–59 (*p* < 0.001). Moreover, there was a statistically significant association between age and epithelial lesions (*p* < 0.001). Figure 3 illustrates the distribution of lesions age‐wise.

As shown in Tables 1 and 2, soft tissue tumors were found mostly in anterior regions (*p* < 0.001) and epithelial lesions in posterior regions of the gingiva (*p* = 0.004). Also, most cases affecting the whole arch or both jaws were either a hematologic or mucocutaneus disease (*p* < 0.001). The latter also had a tendency to the maxillary gingiva (*p* < 0.001). In 16 cases, the affected jaw was not reported, and in 53 cases, the involved region was not documented. Some of these lesions were also found in other sites rather than the gingiva. The most to least common secondary sites were alveolar mucosa (2.8%, *N* = 19), palate (2.3%, *N* = 16), buccal mucosa (1%, *N* = 7), lips (0.4%, *N* = 3), floor of the mouth (0.3%, *N* = 2), skin (0.3%, *N* = 2), and retromolar pad (0.1%, *N* = 1).

**Table 1:** Prevalence of gingival lesions based on the affected jaw.

Group	Maxilla	Mandible	Both	Total
Soft tissue tumors	233 (48.3%)	248 (51.5%)	1 (0.2%)	482
Epithelial lesions	35 (45.5%)	39 (50.6%)	3 (3.9%)	77
Inflammatory lesions	30 (47.6%)	33 (52.4%)	0	63
Mucocutaneous diseases	22 (71%)	5 (16.1%)	4 (12.9%)	31
Hematologic diseases	4 (33.3%)	6 (50%)	2 (16.7%)	12
Peripheral odontogenic lesions	2 (66.7%)	1 (33.3%)	0	3
Total	326 (48.8%)	332 (49.7%)	10 (1.5%)	646

**Table 2:** Prevalence of gingival lesions based on the involved region.

Group	Anterior	Posterior	Both	Total
Soft tissue tumors	268 (58.3%)	191 (41.5%)	1 (0.2%)	460
Epithelial lesions	23 (33.8%)	45 (66.2%)	0	68
Inflammatory lesions	29 (48.3%)	29 (48.3%)	2 (3.3%)	60
Mucocutaneous diseases	12 (41.4%)	14 (48.3%)	3 (10.3%)	29
Hematologic diseases	2 (18.2%)	6 (54.5%)	3 (27.3%)	11
Peripheral odontogenic lesions	0	3 (100%)	0	3
Total	334 (52.9%)	288 (45.6%)	9 (1.4%)	631

Concerning the type of biopsy, incisional biopsy was performed on mucocutaneous lesions and inflammatory lesions such as parulis and foreign body reaction. Other lesions in this study were sent to the pathology department using an excisional biopsy.

The most common specific lesions observed in the study, in descending order, were PGCG with 145 cases (21.2%), PG with 132 cases (19.3%), POF with 122 cases (17.8%), and FFH with 52 cases (7.6%), all of which were soft tissue tumors. PG and PGCG were reported mostly in women (*p* = 0.002 and *p* = 0.007, respectively). Table 3 shows the prevalence of every observed lesion in detail. POF mostly affected younger patients (mean age = 30.4 years), and then PG (mean age = 35.7), PGCG (mean age = 35.9), and FFH (mean age = 36.2). Additionally, a significant correlation between POF and age was identified in this investigation (*p* < 0.001).

**Table 3:** Prevalence of specific gingival lesions found in this study based on age and gender.

Group	Lesion	Prevalence, *N* (%)	M:F	Age (Mean ± SD)
Soft tissue tumors (72.1%)	Peripheral giant cell granuloma	145 (21.2%)	70:75	35.9 ± 19.2
	Pyogenic granuloma	132 (19.3%)	34:98	35.7 ± 17.9
	Peripheral ossifying fibroma	122 (17.8%)	50:72	30.4 ± 14.8
	Focal fibrous hyperplasia	52 (7.6%)	15:37	36.2 ± 16.4
	Giant cell fibroma	18 (2.6%)	6:12	30 ± 17
	Neurofibroma	8 (1.2%)	6:2	27.9 ± 17.5
	Gingival fibromatosis	6 (0.9%)	3:3	19.8 ± 14.8
	Oral focal mucinosis	4 (0.6%)	1:3	18.5 ± 9.5
	Other^a^	5 (0.7%)	1:5	—
Epithelial lesions (11.3%)	Squamous cell carcinoma	32 (4.7%)	17:15	61.7 ± 16.9
	Epithelial hyperplasia with dysplasia	18 (2.6%)	5:13	61.7 ± 16.3
	Squamous papilloma	7 (1%)	2:5	24 ± 12.8
	Malignant melanoma	4 (0.6%)	2:2	45.7 ± 20
	Benign hyperkeratosis	3 (0.4%)	1:2	45 ± 14.52
	Verrucous hyperplasia	3 (0.4%)	0:3	57.7 ± 19.9
	Epithelial hyperplasia	3 (0.4%)	2:1	53.3 ± 18.7
	Other^b^	7 (1%)	2:5	—
Inflammatory lesions (9.6%)	Gingivitis	25 (3.7%)	7:18	33.9 ± 18.4
	Chronic periodontitis	13 (1.9%)	4:9	36.1 ± 14.7
	Foreign body reaction	6 (0.9%)	1:5	43.5 ± 17.6
	Desquamative gingivitis	4 (0.6%)	0:4	45.5 ± 20.4
	Parulis	3 (0.4%)	2:1	22 ± 6.2
	Other^c^	15 (2.2%)	2:13	—
Dermatologic diseases (4.8%)	Oral lichen planus	19 (2.8%)	5:14	51.7 ± 12.8
	Pemphigus vulgaris	5 (0.7%)	2:3	54 ± 8.3
	Lichenoid reaction	4 (0.6%)	0:4	48.5 ± 12.8
	Mucous membrane pemphigoid	4 (0.6%)	0:4	52 ± 21.6
	Bullous pemphigoid	1 (0.1%)	1:0	43
Hematologic disorders (1.8%)	Langerhans cell histiocytosis	8 (1.2%)	6:2	28.4 ± 14.1
	Non‐Hodgkins's lymphoma	3 (0.4%)	2:1	30.7 ± 19.9
	Malignant Lymphoproliferative lesion	1 (0.1%)	1:0	26
Peripheral odontogenic tumors (0.4%)	Peripheral odontogenic fibroma	1 (0.1%)	0:1	30
	Peripheral ameloblastoma	1 (0.1%)	0:1	50
	Eruption cyst	1 (0.1%)	0:1	9
Total		684 (100%)	250:434	37 ± 18.9

^a^ Soft tissue tumor with *N* < 3: malignant small round cell tumor (*N* = 2), benign fibrous histiocytoma, palisaded encapsulated neuroma, and juvenile aggressive fibromatosis, metastatic epithelioid sarcoma (*N* = 1).

^b^ Epithelial lesions with *N* < 3: verrucous carcinoma, undifferentiated carcinoma (*N* = 2), verruca vulgaris, oral melanoacanthoma, and oral melanotic macule (*N* = 1).

^c^ Inflammatory lesions with *N* < 3: aggressive periodontitis, exogenous pigmentation, amalgam tattoo, plasma cell gingivitis, abscess (*N* = 2), pyostomatitis vegetans, drug reaction, sarcoidosis, wegener granulomatosis, and localized juvenile spongiotic gingival hyperplasia (*N* = 1).

Within epithelial lesion, squamous cell carcinoma (SCC) was the most prevalent lesion (4.7%) and also the most common malignancy detected in the gingiva. A higher incidence of SCC was observed in men, accounting for 17 cases (53.1%).

Although clinical features were not mentioned in majority of the lesions, soft tissue tumors were described thoroughly by the clinician. Clinical features like color and consistency in four of the most prevalent lesions are shown in the Table 4. PGCG is mostly purple, POF and FFH are white, and PG is mostly a red lesion. Also, bleeding is mostly associated with PGCG.

**Table 4:** Distribution of clinical features in the most prevalent lesions.

Clinical features	PGCG (%)	PG (%)	POF (%)	FFH (%)
Base				
Pedunculated	62.1	87.9	72.7	66.7
Sessile	37.9	12.1	27.3	33.3
Color				
White	0.0	0.0	5.7	2.9
Pink	11.0	21.6	51.4	62.9
Red	26.8	45.9	22.9	22.9
Purple	57.3	19.7	20.0	11.4
Black	4.9	0.0	0.0	0.0
Consistency				
Elastic	39.4	41.7	31.7	20.0
Soft	15.9	19.2	6.8	6.0
Firm	42.4	36.7	57.3	70.0
Hard	2.3	0.0	4.3	4.0
Ulceration				
Yes	11.4	9.2	9.2	0.0
No	88.6	90.8	90.8	100.0
Bleeding				
Yes	19.3	16.8	9.2	1.9
No	80.7	83.2	90.8	98.1

Abbreviations: FFH, focal fibrous hyperplasia; PG, pyogenic granuloma; PGCG, peripheral giant cell granuloma; POF, peripheral ossifying fibroma.

Size of the lesions was ranged between 0.2 and 4.5 cm. Two lesions had a size of 4.5 cm reportedly, one PG and one POF, both on the maxillary arch. Hematologic lesions had the biggest (1.5 ± 0.5) and mucocutaneous lesions had the smallest mean size (0.9 ± 0.5). There was statistically significant difference between groups means determined by one‐way analysis of variance (*p* = 0.006).

## 4 Discussion

Gingiva is one of the primary regions where oral lesions predominantly form. Categorizing these lesions, understanding their distribution within Iranian population, and comparing these data with existing studies globally, can contribute to improved diagnosis and comprehension of these lesions.

One of the main problems in comparing relevant studies is the lack of a practical and unanimous categorization. Numerous studies classified lesions mainly as either neoplastic or nonneoplastic (Manjunatha et al. 2014; Kamath, Vidya, and Anand 2013; Carbone et al. 2012; Shamim et al. 2008; Orikpete and Iyogun 2021), We find this classification to be less advantageous since it primarily emphasizes the aspect of malignancy. Multiple investigation reported their data based on nonplaque‐induced gingival diseases' classification proposed by Holmstrup et al. (Gupta et al. 2022; Li et al. 2021; Hernández‐Ríos et al. 2018; Holmstrup, Plemons, and Meyle 2018). The issue with this classification lies in its lack of practicality due to its etiology‐based categorization. Lastly, in this study, we designed our groups based on Neville's Oral and Maxillofacial Pathology (Neville et al. 2016), with slight modifications, almost similar to Montazer Lotf‐Elahi, Farzinnia, and Jaafari‐Ashkavandi (2022). We believe that classifying gingival lesion based on a histopathologic similarity can give the reader a better perspective. This is due to the fact that the presentation of lesions within a group, are more alike.

In our study, 662 lesions were observed on the gingiva, accounting for 8.9% of the total examined samples. Other studies reported 3.2% in India (Gupta et al. 2022), 6.23% in Greece (Tamiolakis et al. 2018), 6.7% in Israel (Buchner, Shnaiderman‐Shapiro, and Vered 2010), 9.5% in Saudi Arabia (Alblowi and Binmadi 2018), 14.6% in Nigeria (Orikpete and Iyogun 2021) 15% in the United States (Dovigi et al. 2016), 11.9% (Moridani, Shaahsavari, and Bagher 2014), and 18.9% in Iran (Montazer Lotf‐Elahi, Farzinnia, and Jaafari‐Ashkavandi 2022), and 38.9% in Turkey (Sengüven et al. 2015). This disparity could potentially arise from geographical differences and variations in the study duration or criteria. Another reason could be the distinction of gingival lesion from the lesions on the alveolar mucosa in our study. Furthermore, a higher occurrence was observed in women. This is in line with the results of other studies (Alblowi and Binmadi 2018; Gupta et al. 2022; Tamiolakis et al. 2018; Manjunatha et al. 2014; Hernández‐Ríos et al. 2018; Kamath, Vidya, and Anand 2013; Carbone et al. 2012; Shamim et al. 2008; Orikpete and Iyogun 2021) However, Gupta et al. (2022) reported a higher prevalence in men. The likely reason for this discrepancy might be the small sample size in this study. The mean age of gingival lesions in this study was 37 ± 18.9 years and the highest number of cases were observed in the age group of 20–39 years, which is consistent with most of the reviewed studies (Alblowi and Binmadi 2018; Gupta et al. 2022; Li et al. 2021; Montazer Lotf‐Elahi, Farzinnia, and Jaafari‐Ashkavandi 2022; Shamim et al. 2008; Orikpete and Iyogun 2021; Ababneh 2006). However, Hernández‐Ríos et al. (2018) reported the most presence of gingival lesions in older individuals, with the majority in sixth decade of life, which could potentially be attributed to geographical differences.

### 4.1 Groups

Soft tissue tumors constituted the most prevalent group in this study, which is consistent with other studies (Alblowi and Binmadi 2018; Gupta et al. 2022; Li et al. 2021; Montazer Lotf‐Elahi, Farzinnia, and Jaafari‐Ashkavandi 2022; Hernández‐Ríos et al. 2018; Moridani, Shaahsavari, and Bagher, 2014). Following this, epithelial lesion and inflammatory lesions were ranked next. In the studies by Alblowi and Binmadi (2018) and Gupta et al. (2022) inflammatory lesions were placed second in terms of prevalence. This discrepancy might arise from the fact that our study considered SCC within the group of epithelial lesions, whereas in most studies, it was placed within the malignant lesion category.

### 4.2 Lesions

In this study, PGCG was reported as the most common lesion found on the gingiva. Another study in Iran (Naderi, Eshghyar, and Esfehanian 2012) confirmed our results. However, most of the studies reported either PG or FFH as the most prevalent lesions. Table 5 compares our results to other studies. It's worth mentioning that in most studies, either PGCG or POF exhibited the lowest prevalence among these lesions. These variations might be attributed to racial and geographical variations or to different histopathologic criteria. Moreover, it could be due to the fact that we specifically excluded lesions on the alveolar mucosa while other studies did not take that into application.

**Table 5:** Most common reported soft tissue tumors in the past studies.

	Soft tissue tumors			
	PGCG	PG	POF	FFH
Studies	Iran, 2012 (Naderi, Eshghyar, and Esfehanian 2012) Current study, Iran, 2023	Iran, 2007 (Zarei, Chamani, and Amanpoor 2007) India, 2008 (Shamim et al. 2008) India, 2012 (Kashyap, Reddy, and Nalini 2012) India, 2013 (Kamath, Vidya, and Anand 2013) India, 2014 (Manjunatha et al. 2014) Saudi Arabia, 2018 (Alblowi and Binmadi 2018) Greece, 2018 (Tamiolakis et al. 2018) Nigeria, 2021 (Orikpete and Iyogun 2021) Iran, 2022 (Montazer Lotf‐Elahi, Farzinnia, and Jaafari‐Ashkavandi 2022)	None	Israel, 2010 (Buchner, Shnaiderman‐Shapiro, and Vered 2010) Italy, 2012 (Carbone et al. 2012) Turkey, 2013 (Sengüven et al. 2015) Chile, 2018 (Hernández‐Ríos et al. 2018) India, 2020 (Lakkam et al. 2020) China, 2022 (Li et al. 2021) India, 2022 (Gupta et al. 2022) Brazil, 2023 (Baesso et al. 2023)

Abbreviations; FFH, focal fibrous hyperplasia; PG, pyogenic granuloma; PGCG, peripheral giant cell granuloma; POF, peripheral ossifying fibroma.

**Figure 4:**
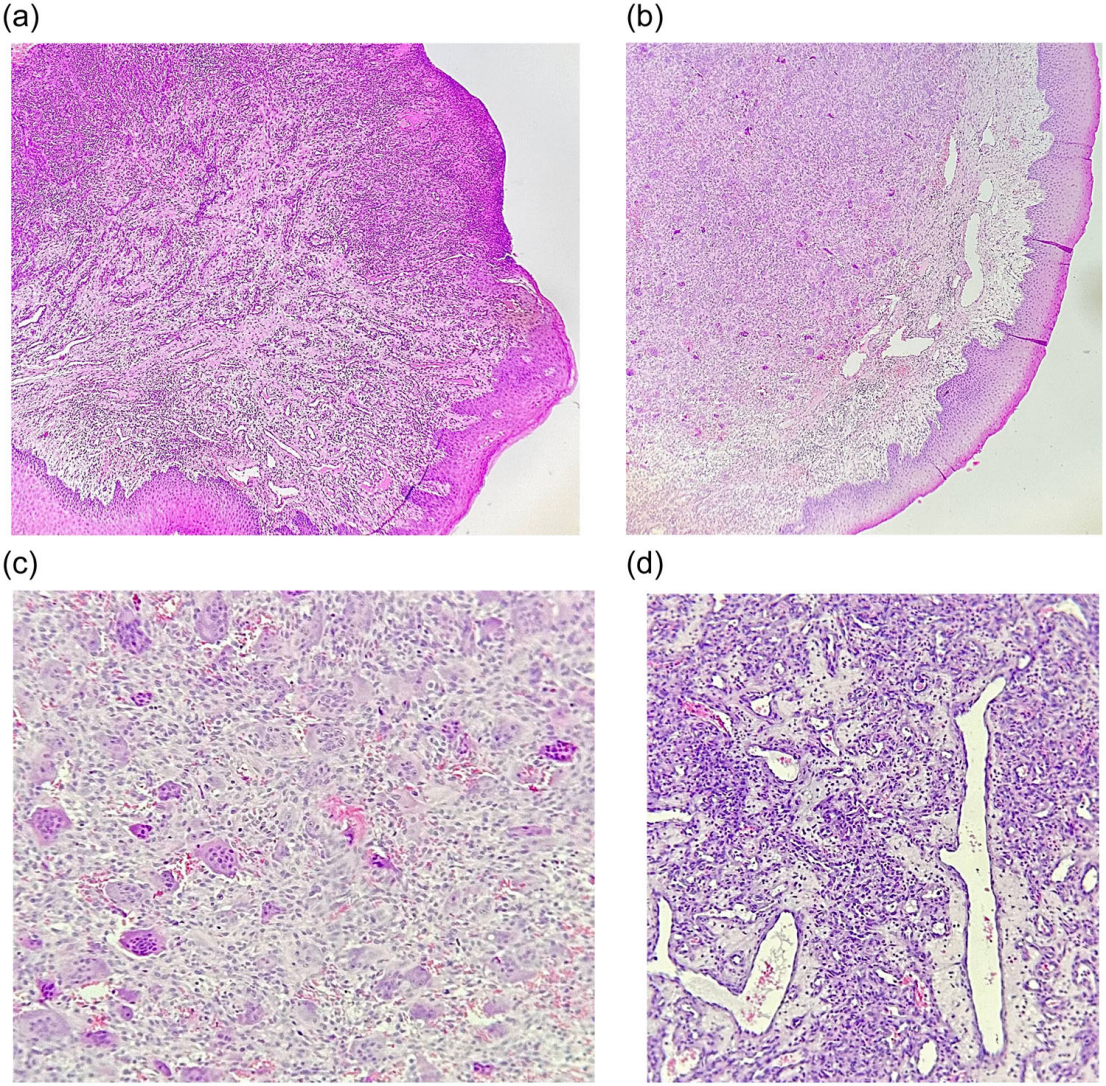
(a) Low‐power view showing an exophytic mass of granulation tissue with an ulcerated surface, (b) low‐power view showing a nodular prefiltration of multinucleated giant cells within the gingiva, (c) high‐power view showing scattered giant cells within a hemorrhagic background of ovoid and spindle‐shaped mesenchymal cells, and (d) higher‐power view showing capillary blood vessels and scattered inflammation.

As the majority of the studies, we also did an in‐depth comparison of the noted lesions. Moreover, histopathologic images of some of these lesions were provided in Figure 4 for better understanding. PGCG, PG, POF, and FFH were all shown a female predominance in most of the previous studies, same as ours. However, some studies showed a higher prevalence towards men in PGCG (Gupta et al. 2022; Dutra et al. 2019; Buchner, Shnaiderman‐Shapiro, and Vered 2010; Baesso et al. 2023; Zarei, Chamani, and Amanpoor 2007) which had the most diversity, PG (Alblowi and Binmadi 2018; Naderi, Eshghyar, and Esfehanian 2012), POF (Gupta et al. 2022; Kashyap, Reddy, and Nalini 2012) or in FFH (Gupta et al. 2022). Regarding clinical features, Baesso et al. (2023). reported that these four lesions are mostly red, and also, FFH is mostly sessile. However, in this study, only PG was found to be mostly red, and FFH was also reported as pedunculated.

In the current study, these lesions were reported with mean ages falling within the fourth decade of life. There was a tendency to younger patients in POF and then in an ascending order in PG, PGCG, and FFH. A few studies showed an almost similar pattern (Naderi, Eshghyar, and Esfehanian 2012) except that in some of them, patients with FFH were younger compared to PGCG (Montazer Lotf‐Elahi, Farzinnia, and Jaafari‐Ashkavandi 2022; Dovigi et al. 2016). In contrast, Tamiolakis et al. (2018), Lakkam et al. (2020), and Baesso et al. (2023) observed higher age preferences for any of these lesions. Also, Dutra et al. (2019), Naderi, Eshghyar, and Esfehanian (2012), and Zarei, Chamani, and Amanpoor (2007) reported PGCG in relatively younger ages.

SCC was the most prevalent epithelial, and also malignant lesion in our study, which was consistent with other studies (Alblowi and Binmadi 2018; Li et al. 2021; Montazer Lotf‐Elahi, Farzinnia, and Jaafari‐Ashkavandi 2022; Manjunatha et al. 2014; Kamath, Vidya, and Anand 2013; Carbone et al. 2012) However, Hernández‐Ríos et al. (2018) reported Benign hyperkeratosis as the most common epithelial lesion. We observed that SCC was more frequent in men which was in line with most of the previous studies (Alblowi and Binmadi 2018; Gupta et al. 2022; Montazer Lotf‐Elahi, Farzinnia, and Jaafari‐Ashkavandi 2022; Martínez et al. 2016; Kamath, Vidya, and Anand 2013; Carbone et al. 2012; Dovigi et al. 2016). Shamim et al. (2008) reported a M:F ratio of 1:1 and Tamiolakis et al. (2018) identified a female predominance in SCC lesions, differing from our results.

Gingival lichen planus comprised 2.8% of all the lesions. Lichen planus was more common in women in their 6th decade of life. This was in line with other studies (Mignogna, Russo, and Fedele 2005; Keller and Lombardi 2023). Moreover, 68.4% of these lesions presented a mixed form. Other studies reported the same (Mignogna, Russo, and Fedele 2005; Keller and Lombardi 2023).

## 5 Conclusion

The present study showed that soft tissue tumors are the most common group of lesions found in the gingiva and all of the groups except hematologic lesions had a higher prevalence in women. PGCG, PG, POF, and FFH were the most frequent biopsied lesions, with mean ages in the fourth decade of life. However, POF tended to affect relatively younger patients. Moreover, SCC was the most prevalent malignant lesion. In conclusion, it is advisable to conduct more extensive studies to assess gingival lesions on a larger scale and at 5‐year intervals to monitor any shifts in their prevalence among the population.

## Author Contributions

**Nafiseh Shamloo:** conceptualization, methodology, project administration, supervision, writing—review and editing; **Mostafa Alam:** investigation, resources, writing—review and editing; **Armin Khaleghi:** conceptualization, data curation, formal analysis, investigation, validation, writing—original draft.

## Conflicts of Interest

The authors declare no conflicts of interest.

## Data Availability

The data that support the findings of this study are available on request from the corresponding author. The data are not publicly available due to privacy or ethical restrictions.
